# Bacterial pathogen spectrum and antimicrobial resistance in positive pus cultures from hospitalized patients at a maternal and child healthcare specialty hospital in Shenzhen, China, 2021–2025

**DOI:** 10.3389/fcimb.2026.1860912

**Published:** 2026-06-15

**Authors:** Tongyan Ding, Xiaochun Liu, Futing Liao, Shaoxiang Lin, Shuyan Liu, Shujuan Yang, Xiaojie Zhou, Qiaoxin Zhang, Zhenwen Zhou

**Affiliations:** 1 Clinical Laboratory, Key Laboratory of Bacterial Antimicrobial Resistance and Prevention, Medical Research Institute of Maternal and Child, Longgang District Maternity & Child Healthcare Hospital of Shenzhen City (Affiliated Shenzhen Women and Children’s Hospital (Longgang) of Shantou University Medical College), Shenzhen, Guangdong, China; 2 Clinical Laboratory, Guangzhou Women and Children’s Medical Center, Guangzhou Medical University, Guangdong Provincial Clinical Research Center for Laboratory Medicine, Guangzhou, Guangdong, China; 3 The First Affiliated Hospital of Shantou University Medical College, Shantou, Guangdong, China

**Keywords:** antimicrobial susceptibility profile, *Escherichia coli*, maternal and child healthcare specialty hospital, pathogens, pus culture

## Abstract

**Objective:**

Pus culture and antimicrobial susceptibility testing play an important role in pathogen identification and the adjustment of empirical therapy for localized suppurative infections. This study aimed to investigate the distribution of major bacterial pathogens isolated from pus cultures and their susceptibility profiles to commonly used antimicrobial agents among hospitalized patients at Longgang District Maternity & Child Healthcare Hospital of Shenzhen City, in order to provide evidence for rational clinical antimicrobial use.

**Methods:**

We retrospectively collected pus culture records and aggregated antimicrobial susceptibility data from January 1, 2021 to December 31, 2025.

**Results:**

From 2021 to 2025, 586 non-duplicate bacterial isolates were recovered from positive pus cultures, of which 64.7% were Gram-negative and 35.3% were Gram-positive. The leading isolates were *Escherichia coli* (48.0%) and *Staphylococcus aureus* (22.5%), followed by *Klebsiella pneumoniae subsp. pneumoniae* (7.0%), *Staphylococcus epidermidis* (3.8%), Pseudomonas aeruginosa (2.4%), and *Streptococcus agalactiae* (1.4%). Positive cultures were mainly from the Departments of Pediatric Surgery, General Surgery, Gynecology, and Breast Surgery, with a bimodal age distribution peaking in young children and women of reproductive age; among Pediatric Surgery cases, males predominated, especially in the 0-3-year group. Extended-spectrum beta-lactamase production was detected in 28.1% of *Escherichia coli* and 20.0% of *Klebsiella pneumoniae subsp. pneumoniae* isolates. *Escherichia coli* showed high resistance to ampicillin, trimethoprim-sulfamethoxazole, and ciprofloxacin but remained highly susceptible to imipenem and amikacin. *Klebsiella pneumoniae subsp. pneumoniae* remained highly susceptible to amikacin, cefoxitin, and ertapenem despite resistance to several cephalosporins and ciprofloxacin. A subset of *Staphylococcus aureus* isolates was methicillin resistant, with frequent resistance to erythromycin and clindamycin, whereas vancomycin and linezolid retained good *in vitro* activity.

**Conclusions:**

Empirical antimicrobial therapy for localized suppurative infections in this maternal and child healthcare specialty hospital should be guided by local pathogen distribution and antimicrobial susceptibility surveillance, with coverage for both Enterobacterales and staphylococci.

## 1 Introduction

Pus culture specimens are typically obtained from abscesses, postoperative incision infections, skin and soft tissue infections, and other localized infectious lesions accompanied by purulent discharge. As such, their microbiological findings can, to some extent, reflect the pathogen spectrum of localized suppurative infections in hospitalized patients (Mansoor et al., 2024; Muttiah and Hanafiah, 2025; Rezaei et al., 2025). Although these infections often present as localized lesions, their pathogen profiles are influenced by multiple factors, including the site of infection, host characteristics, perioperative management, prior antimicrobial exposure, and healthcare-related exposures, resulting in considerable clinical heterogeneity (Bucataru et al., 2023; Rizzo et al., 2025; Toschi et al., 2025). In clinical practice, once incisional suppuration, localized abscess formation, or increased purulent discharge occurs, antimicrobial treatment often needs to be initiated promptly. However, treatment success depends not only on the timely use of antimicrobial agents, but also on whether the empirical regimen adequately covers the causative pathogens and whether therapy can subsequently be adjusted in a timely manner according to culture and susceptibility results (Allaw et al., 2025; Blanes Hernández et al., 2023). Therefore, the value of pus culture and antimicrobial susceptibility testing extends beyond laboratory pathogen detection; more importantly, it provides direct evidence for pathogen identification, optimization of empirical therapy, and antimicrobial stewardship in localized suppurative infections.

Unlike blood or urine specimens, which are relatively uniform in origin, pus specimens arise from a broader range of clinical settings, including abscesses, postoperative wound infections, drainage fluid submissions, and other localized purulent lesions (Almohrij, 2025). This complexity in specimen origin means that pus culture results are not confined to a single infection type, but instead can provide a more integrated picture of the microbiological distribution across a variety of localized suppurative infections within a hospital. For this reason, although the interpretation of pus culture data is inherently more challenging, such data also offer a distinct advantage not fully shared by specimens from a single organ system, namely, the ability to reflect the microbiological characteristics of localized infections shaped by different inpatient populations at the institutional level.

In recent years, the empirical management of localized suppurative infections has become increasingly challenging because of the widespread use of antimicrobial agents, the growing frequency of perioperative interventions, and the continuing evolution of antimicrobial resistance (Blackburn et al., 2025). Traditionally, purulent lesions have been viewed primarily as infections dominated by Gram-positive cocci, especially *Staphylococcus aureus* (Giudice, 2020; Linz et al., 2023; Reyes Ruiz et al., 2024). However, in real-world inpatient settings, Gram-negative bacilli, particularly members of the Enterobacterales, also play an important role in many localized infections (Bargavi et al., 2024; Hamudu et al., 2025; Ramesh et al., 2025). In complex wounds, perioperative infections, and some patients with prolonged hospitalization, more difficult opportunistic pathogens may also be encountered (Mukomena et al., 2024; Roy et al., 2024). These observations indicate that localized suppurative infections do not correspond to a fixed pathogen spectrum. Rather, their microbiological composition is highly dependent on patient source, inpatient specialty structure, infection context, and the local hospital microbiological environment.

Previous domestic and international studies on pus or wound infection cultures also support this view. Studies involving pediatric infections, women-related purulent lesions, and general wound infections in China have suggested that *Staphylococcus aureus*, *Escherichia coli*, *Pseudomonas aeruginosa*, *Klebsiella pneumoniae*, and *Acinetobacter baumannii* are common isolates from pus or wound infections, with resistance concerns including methicillin-resistant Staphylococcus aureus, extended-spectrum *β*-lactamase-producing Enterobacterales, and multidrug-resistant Gram-negative bacteria (Jia et al., 2025; Khan et al., 2025; Wang et al., 2024). International studies on pus or wound infections have reported similar pathogen distributions and resistance challenges (Khalid et al., 2024; Mansoor et al., 2024). These findings are broadly consistent with global antimicrobial resistance trends, in which Staphylococcus aureus, *Escherichia coli*, *Klebsiella pneumoniae*, *Acinetobacter baumannii*, and *Pseudomonas aeruginosa* remain important contributors to the global burden of bacterial infections and antimicrobial resistance (Antimicrobial Resistance Collaborators, 2022). However, systematic data on pus culture isolates from hospitalized patients across multiple departments in maternal and child healthcare specialty hospitals remain limited. Therefore, local surveillance of the pathogen spectrum and antimicrobial susceptibility profiles of pus culture isolates may help supplement the etiological evidence for localized suppurative infections in this specific healthcare setting and provide context-specific evidence for empirical antimicrobial selection and antimicrobial stewardship.

Compared with general hospitals, maternal and child healthcare specialty hospitals have more distinctive specialty-specific characteristics with respect to the pathogen spectrum and treatment decision-making associated with localized suppurative infections (Wong et al., 2023). On the one hand, their inpatient population is not limited to obstetric patients, but also includes gynecologic, general surgical, pediatric surgical, and other perioperative pediatric and female patients. As a result, pus specimens often show a marked cross-specialty distribution. On the other hand, antimicrobial selection in pregnant women, women of reproductive age, and children must balance efficacy with safety, and some agents commonly used in the treatment of infections in the general adult population are more restricted in these special populations (Bailey et al., 2025; Nguyen et al., 2025). In other words, antimicrobial treatment in maternal and child healthcare specialty hospitals must address not only whether an agent is effective, but also whether it is appropriate for a specific patient group. This dual requirement further increases the value of local pathogen surveillance and cumulative susceptibility data in such institutions, as these data directly influence the accuracy and feasibility of empirical therapy and the scientific basis of antimicrobial stewardship.

Against this background, the present study retrospectively analyzed pus culture submissions and corresponding antimicrobial susceptibility results from hospitalized patients at Longgang District Maternity & Child Healthcare Hospital of Shenzhen City between 2021 and 2025. From the perspectives of major pathogen composition and susceptibility to commonly used antimicrobial agents, we sought to systematically characterize the pathogen spectrum and resistance patterns associated with localized suppurative infections at our institution. We hope that this analysis will provide laboratory evidence more closely aligned with real-world clinical practice for empirical antimicrobial treatment of localized suppurative infections in our hospital, while also generating localized data to support antimicrobial stewardship and continued institutional pathogen surveillance in maternal and child healthcare specialty hospitals.

## 2 Materials and methods

### 2.1 Study design and data source

This was a single-center, retrospective descriptive study. Study data were obtained from the laboratory information system of Longgang District Maternity & Child Healthcare Hospital of Shenzhen City, covering the period from January 1, 2021 to December 31, 2025. Based on pus culture–related records exported from the system, we collected data on specimen submissions, culture results, basic patient characteristics, submitting departments, and aggregated antimicrobial susceptibility test results. These data were used to analyze the distribution of pathogens isolated from pus cultures and the antimicrobial susceptibility patterns of major isolates among hospitalized patients. The study was approved by the Research Project Ethics Committee of Longgang District Maternity & Child Healthcare Hospital of Shenzhen City (approval no. LGFYKYXMLL-2026-2). As this study was based on previously collected anonymized laboratory data and involved neither additional intervention nor identifiable patient information, the requirement for informed consent was waived.

### 2.2 Study population and data processing

This study included pus culture records from hospitalized patients. Only pus or purulent secretion specimens submitted for routine diagnostic bacterial culture were included. Surveillance or screening specimens, non-pus specimens, and duplicate isolates were excluded based on the specimen type and test item recorded in the laboratory information system. Because the original database did not provide standardized and complete information on infection sites or specific disease categories, no further stratification by anatomical site or clinical diagnosis was performed, and pus culture specimens were analyzed as a whole. To minimize the effect of repeated submissions on the analysis of bacterial distribution, only the first non-duplicate isolate was included when the same patient had repeated submissions or when the same pathogen was repeatedly isolated. Records with no bacterial growth, no pathogenic bacteria recovered, or reports indicating “more than three bacterial species isolated (suspected contamination; repeat sampling recommended)” were excluded from the analysis of positive pathogen distribution.

### 2.3 Pus specimen collection and culture

Pus specimens were collected as part of routine clinical care by trained clinicians using aseptic techniques. When possible, purulent material was aspirated from abscesses or localized purulent lesions using a sterile syringe after appropriate skin disinfection; wound or incision secretions were collected using sterile swabs when aspiration was not feasible. Specimens were placed in sterile containers or appropriate transport media and sent promptly to the clinical microbiology laboratory for processing. Upon receipt, specimens were inoculated onto routine culture media, including blood agar, chocolate agar, and MacConkey agar, according to standard laboratory procedures. Culture plates were incubated at 37 °C for 24 h under appropriate atmospheric conditions, and incubation was extended when necessary according to colony growth. Suspected pathogenic colonies were selected for further bacterial identification and antimicrobial susceptibility testing.

### 2.4 Bacterial identification and antimicrobial susceptibility testing

Bacterial identification and antimicrobial susceptibility testing were performed using the VITEK 2 Compact automated microbiology system (bioMérieux, France). Antimicrobial susceptibility results were extracted from routine clinical laboratory reports and were interpreted according to the CLSI criteria in use by the clinical microbiology laboratory at the time of testing. The 2025 CLSI breakpoints were not retrospectively applied to all historical isolates collected during 2021-2025. For Enterobacterales, extended-spectrum *β*-lactamase (ESBL) production was determined based on the ESBL detection results generated by the VITEK 2 Compact system as part of routine clinical laboratory testing. Additional manual phenotypic confirmatory testing was not routinely performed for all isolates in this retrospective dataset. According to the antimicrobial panels available for different bacterial species, the resistance, susceptibility, and intermediate rates of major clinically relevant isolates to commonly used antimicrobial agents were calculated separately.

### 2.5 Quality control

Quality control was implemented throughout the laboratory workflow in accordance with the relevant requirements of CLSI M100, 35th edition (2025). Standard reference strains, including *Escherichia coli* ATCC 25922, *Klebsiella pneumoniae* ATCC 700603, *Staphylococcus aureus* ATCC 25923, *Enterococcus faecalis* ATCC 29212, and *Streptococcus pneumoniae* ATCC 49619, were used for routine quality control of bacterial identification and antimicrobial susceptibility testing to ensure the accuracy and reproducibility of the results.

### 2.6 Study variables

The main variables analyzed in this study included: (1) the sex, age, and submitting department of the included cases; (2) the distribution of major pathogens isolated from positive pus cultures; and (3) the susceptibility, resistance, and intermediate rates of major isolates to commonly used antimicrobial agents.

### 2.7 Statistical analysis

This study was primarily descriptive in nature. Categorical variables were presented as counts and percentages. The analysis of pathogen distribution was based on the first non-duplicate isolate, and antimicrobial susceptibility results were summarized and described using aggregated laboratory susceptibility data. Descriptive statistical analysis and figure preparation were performed using Origin 2024 software (OriginLab Corporation, Northampton, MA, USA). Because this study was descriptive, no formal statistical testing for temporal trends or multiple group comparisons was performed; therefore, no significance threshold or multiple-comparison adjustment was applied.

## 3 Results

### 3.1 Bacterial distribution

#### 3.1.1 Distribution of major pathogens isolated from positive pus cultures

From 2021 to 2025, a total of 586 bacterial isolates were recovered from positive pus cultures in our hospital (**Table 1**), of which 379 (64.7%) were Gram-negative and 207 (35.3%) were Gram-positive. Among all isolates, *Escherichia coli* was the predominant pathogen (281, 48.0%), followed by *Staphylococcus aureus* (132, 22.5%), *Klebsiella pneumoniae subsp. pneumoniae* (41, 7.0%), *Staphylococcus epidermidis* (22, 3.8%), *Pseudomonas aeruginosa* (14, 2.4%), and *Streptococcus agalactiae* (8, 1.4%). Other Gram-negative and Gram-positive bacteria accounted for 43 (7.3%) and 45 (7.7%) isolates, respectively. Descriptive year-by-year summary showed that Gram-positive bacteria accounted for 53.1% and 44.9% of isolates in 2021 and 2022, respectively, which was broadly comparable to the proportions of Gram-negative bacteria (46.9% and 55.1%). In 2023, 2024, and 2025, Gram-negative bacteria accounted for 70.5%, 73.0%, and 70.1% of isolates, respectively, whereas Gram-positive bacteria accounted for 29.5%, 27.0%, and 29.9%, respectively.

**Table 1:** Distribution of major bacterial isolates from positive pus cultures, 2021–2025 [n (%)].

Organism	2021 (n=81)		2022(n=118)		2023(n=129)		2024(n=141)		2025(n=117)		Total(n=586)	
	n	%	n	%	n	%	n	%	n	%	n	%
Gram-negative bacteria	38	46.9	65	55.1	91	70.5	103	73.0	82	70.1	379	64.7
*Escherichia coli*	26	32.1	43	36.4	68	52.7	79	56.0	65	55.6	281	48.0
*Klebsiella pneumoniae subsp. pneumoniae*	4	4.9	8	6.8	15	11.6	11	7.8	3	2.6	41	7.0
*Pseudomonas aeruginosa*	3	3.7	4	3.4	1	0.8	2	1.4	4	3.4	14	2.4
Other Gram-negative bacteria	5	6.2	10	8.5	7	5.4	11	7.8	10	8.5	43	7.3
Gram-positive bacteria	43	53.1	53	44.9	38	29.5	38	27.0	35	29.9	207	35.3
*Staphylococcus aureus*	31	38.3	40	33.9	21	16.3	21	14.9	19	16.2	132	22.5
*Staphylococcus epidermidis*	3	3.7	6	5.1	7	5.4	5	3.5	1	0.9	22	3.8
*Streptococcus agalactiae*	1	1.2	2	1.7	1	0.8	3	2.1	1	0.9	8	1.4
Other Gram-positive bacteria	8	9.9	5	4.2	9	7.0	9	6.4	14	12.0	45	7.7

With respect to the annual distribution of major species, *Escherichia coli* was the most frequent isolate in each study year, accounting for 32.1% in 2021, 36.4% in 2022, 52.7% in 2023, 56.0% in 2024, and 55.6% in 2025. *Staphylococcus aureus* ranked second in each year, accounting for 38.3% in 2021, 33.9% in 2022, 16.3% in 2023, 14.9% in 2024, and 16.2% in 2025. The proportion of *Klebsiella pneumoniae subsp. pneumoniae* varied between 2.6% and 11.6%, whereas *Pseudomonas aeruginosa* remained relatively uncommon, ranging from 0.8% to 3.7% across study years. *Staphylococcus epidermidis* and *Streptococcus agalactiae* were also detected at low frequencies, accounting for 0.9%-5.4% and 0.8%-2.1%, respectively.

#### 3.1.2 Departmental distribution and age characteristics of patients with positive pus cultures

**Figure 1:**
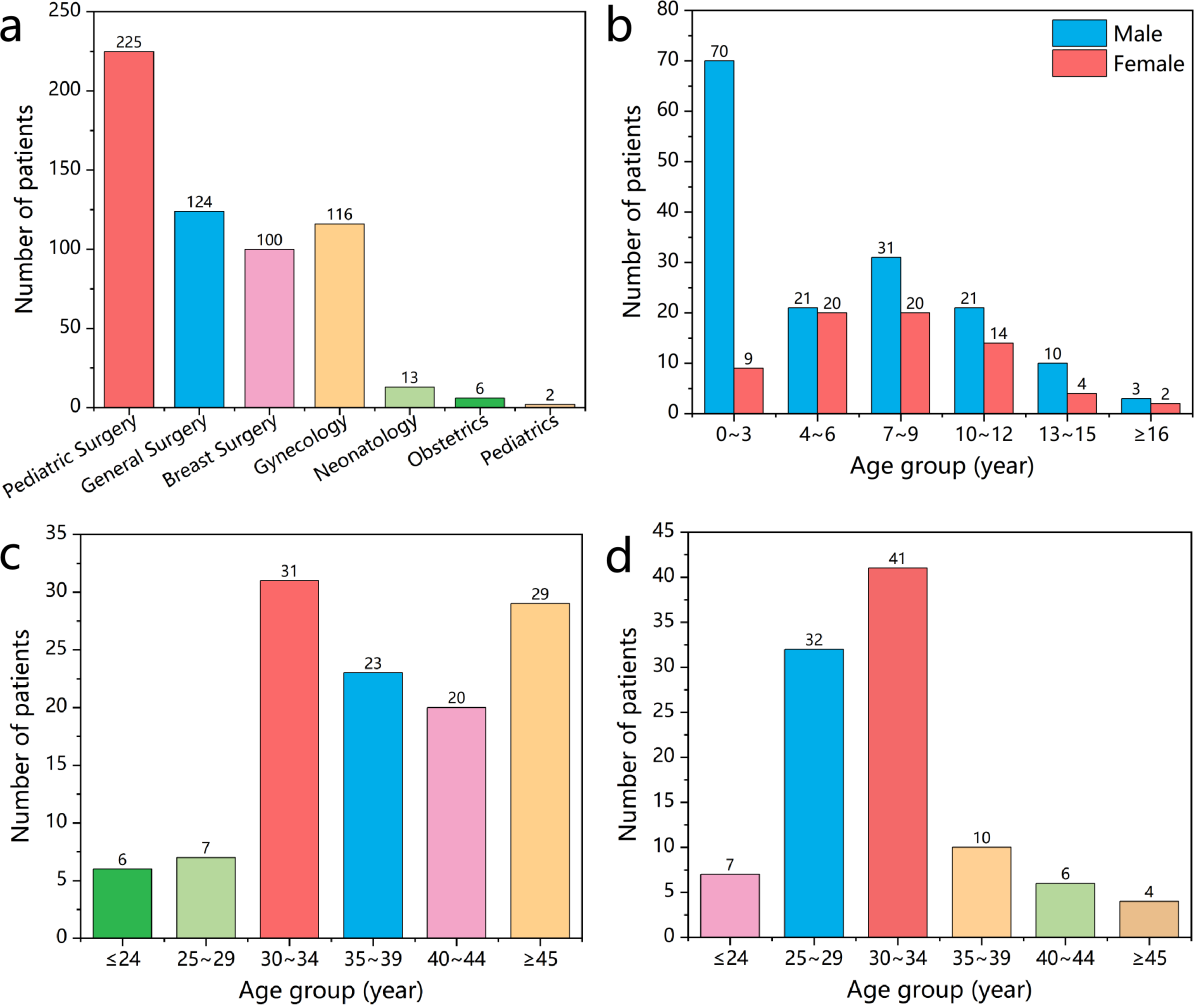
Departmental distribution of positive pus culture specimens and age distribution of patients across different departments. Note: **(a)** departmental distribution of positive pus culture specimens; **(b)** age and sex distribution of patients in the Department of Pediatric Surgery; **(c)** age distribution of patients in the Department of Gynecology; **(d)** age distribution of patients in the Department of Breast Surgery.

Positive pus culture specimens were mainly submitted from the Departments of Pediatric Surgery, General Surgery, Gynecology, and Breast Surgery, with the largest number from Pediatric Surgery (225 cases), followed by General Surgery (124), Gynecology (116), and Breast Surgery (100). In contrast, relatively few cases were from the Departments of Neonatology, Obstetrics, and Pediatrics, with 13, 6, and 2 cases, respectively (**Figure 1a**).

In terms of age distribution, Pediatric Surgery cases were concentrated in the younger age groups, particularly 0–3 years, and declined progressively with increasing age; children aged 4–6 and 7–9 years still accounted for a certain proportion, whereas cases older than 10 years were relatively few (**Figure 1b**). Male patients outnumbered female patients across all age groups, especially in the 0–3-year group. Gynecology cases were mainly distributed among women of reproductive age and older age groups, with the highest number in those aged 30–34 years (n = 31), followed by those aged ≥45 years (n = 29) and 35–39 years (n = 23), while fewer cases were observed in those aged ≤24 and 25–29 years (**Figure 1c**). Breast Surgery cases were concentrated mainly in the 25–34-year age group, with the highest number in those aged 30–34 years (n = 41), followed by those aged 25–29 years (n = 32), and progressively fewer cases in those aged 35 years or older (**Figure 1d**).

### 3.2 Antimicrobial susceptibility profiles of major gram-negative bacilli

#### 3.2.1 Escherichia coli

*Escherichia coli* was the predominant Gram-negative bacillus in this study, with an extended-spectrum beta-lactamase production rate of 28.1% (**Table 2**). Antimicrobial susceptibility testing showed that *Escherichia coli* remained highly susceptible to imipenem and amikacin, with susceptibility rates of 100.0% and 99.3%, respectively. High susceptibility rates were also observed for piperacillin/tazobactam, cefazolin, and cefoxitin, at 97.9%, 97.9%, and 93.1%, respectively. In addition, *Escherichia coli* showed good *in vitro* activity against ceftazidime and cefepime, with susceptibility rates of 86.8% and 84.0%, respectively.

**Table 2:** Antimicrobial resistance profile of Escherichia coli to commonly used antibiotics.

Antimicrobial agents	Resistance rate(%)	Susceptibility rate (%)	Intermediate rate (%)
ESBL screening	28.1	71.9	0.0
Ampicillin	77.9	20.8	1.3
Ampicillin/sulbactam	36.4	35.1	28.6
Piperacillin/tazobactam	2.1	97.9	0.0
Cefazolin	2.1	97.9	0.0
Ceftazidime	8.5	86.8	4.6
Cefepime	10.7	84.0	5.3
Cefoxitin	3.9	93.1	2.9
Imipenem	0.0	100.0	0.0
Amikacin	0.7	99.3	0.0
Ciprofloxacin	28.6	58.4	13.0
Trimethoprim-sulfamethoxazole	47.7	52.3	0.0

#### 3.2.2 *Klebsiella pneumoniae* subsp. *pneumoniae*

The extended-spectrum beta-lactamase production rate of *Klebsiella pneumoniae subsp. pneumoniae* was 20.0% (**Table 3**). Antimicrobial susceptibility testing showed that this organism remained fully susceptible to amikacin, cefoxitin, and ertapenem, with susceptibility rates of 100.0% for all three agents. High susceptibility rates were also observed for piperacillin/tazobactam, amoxicillin/clavulanic acid, trimethoprim-sulfamethoxazole, and ampicillin/sulbactam, at 97.6%, 96.4%, 92.7%, and 92.3%, respectively. In addition, susceptibility to cefotaxime and cefepime was 90.2% for both agents, and susceptibility to ciprofloxacin was 84.6%.

**Table 3:** Antimicrobial resistance profile of *Klebsiella pneumoniae subsp. pneumoniae* to commonly used antibiotics.

Antimicrobial agents	Resistance rate (%)	Susceptibility rate (%)	Intermediate rate (%)
ESBL screening	20.0	80.0	0.0
Amikacin	0.0	100.0	0.0
Amoxicillin/clavulanic acid	3.6	96.4	0.0
Ampicillin/sulbactam	7.7	92.3	0.0
Piperacillin/tazobactam	2.4	97.6	0.0
Cefuroxime sodium	20.7	79.3	0.0
Ceftriaxone	19.5	80.5	0.0
Cefotaxime	4.9	90.2	4.9
Cefepime	4.9	90.2	4.9
Cefoxitin	0.0	100.0	0.0
Ertapenem	0.0	100.0	0.0
Ciprofloxacin	15.4	84.6	0.0
Trimethoprim-sulfamethoxazole	7.3	92.7	0.0

By contrast, resistance was relatively more pronounced to several cephalosporins. The resistance rates to cefuroxime sodium and ceftriaxone were 20.7% and 19.5%, respectively, while resistance to ciprofloxacin was 15.4%. Resistance rates to ampicillin/sulbactam and trimethoprim-sulfamethoxazole were 7.7% and 7.3%, respectively. Intermediate susceptibility was observed for both cefotaxime and cefepime, each at 4.9%.

#### 3.2.3 Pseudomonas aeruginosa

*Pseudomonas aeruginosa* remained highly susceptible to most commonly used antimicrobial agents (**Table 4**). Susceptibility rates to imipenem and meropenem were both 100.0%. High susceptibility rates were also observed for piperacillin/tazobactam, ceftazidime, cefepime, and amikacin, all at 92.9%. In addition, susceptibility rates to ciprofloxacin and levofloxacin were 84.6% and 85.7%, respectively.

**Table 4:** Antimicrobial resistance profile of *Pseudomonas aeruginosa* to commonly used antibiotics.

Antimicrobial agents	Resistance rate (%)	Susceptibility rate (%)	Intermediate rate (%)
Piperacillin/tazobactam	7.1	92.9	0.0
Ceftazidime	7.1	92.9	0.0
Cefepime	0.0	92.9	7.1
Imipenem	0.0	100.0	0.0
Meropenem	0.0	100.0	0.0
Amikacin	7.1	92.9	0.0
Ciprofloxacin	15.4	84.6	0.0
Levofloxacin	14.3	85.7	0.0

In terms of resistance, relatively higher resistance rates were observed for ciprofloxacin (15.4%) and levofloxacin (14.3%). Resistance rates to piperacillin/tazobactam, ceftazidime, and amikacin were each 7.1%. An intermediate rate of 7.1% was observed for cefepime.

### 3.3 Antimicrobial susceptibility profiles of major gram-positive cocci

#### 3.3.1 Staphylococcus aureus

The antimicrobial susceptibility profile of *Staphylococcus aureus* is shown in **Table 5**. The resistance rates of *Staphylococcus aureus* to cefoxitin screening and oxacillin were 25.0% and 22.0%, respectively, indicating the presence of a proportion of methicillin-resistant *Staphylococcus aureus* among pus isolates in our hospital. The highest resistance rate was observed for penicillin (81.8%), followed by erythromycin (48.5%) and clindamycin (47.7%). In contrast, *Staphylococcus aureus* remained highly susceptible to trimethoprim-sulfamethoxazole, ciprofloxacin, and gentamicin, with susceptibility rates of 94.7% for all three agents. Good *in vitro* activity was also observed for vancomycin and linezolid, with susceptibility rates of 100.0% and 99.2%, respectively. In addition, the intermediate rates for clindamycin, ciprofloxacin, and linezolid were 0.8%, 1.3%, and 0.8%, respectively.

**Table 5:** Antimicrobial resistance profile of *Staphylococcus aureus* to commonly used antibiotics.

Antimicrobial agents	Resistance rate (%)	Susceptibility rate (%)	Intermediate rate (%)
Cefoxitin screening	25.0	75.0	0.0
Oxacillin	22.0	78.0	0.0
Penicillin	81.8	18.2	0.0
Erythromycin	48.5	51.5	0.0
Clindamycin	47.7	51.5	0.8
Trimethoprim-sulfamethoxazole	5.3	94.7	0.0
Ciprofloxacin	4.0	94.7	1.3
Gentamicin	2.3	94.7	0.0
Vancomycin	0.0	100.0	0.0
Linezolid	0.0	99.2	0.8

#### 3.3.2 Staphylococcus epidermidis

The antimicrobial susceptibility profile of *Staphylococcus epidermidis* is shown in **Table 6**. Among the 22 recovered isolates, the resistance rate to cefoxitin screening was 68.2%, suggesting a high proportion of cefoxitin-resistant isolates. High resistance rates were also observed for penicillin and erythromycin, at 90.9% and 63.6%, respectively. Resistance to levofloxacin was also noted, with a rate of 31.8%. In contrast, resistance rates to clindamycin, trimethoprim-sulfamethoxazole, ciprofloxacin, and gentamicin were relatively low, at 13.6%, 9.1%, 9.1%, and 4.5%, respectively, corresponding to susceptibility rates of 81.8%, 90.9%, 90.9%, and 86.4%. In addition, all *Staphylococcus epidermidis* isolates were susceptible to vancomycin and linezolid, and no resistant isolates were detected. A small proportion of isolates showed intermediate susceptibility to clindamycin and gentamicin, with rates of 4.5% and 9.1%, respectively. Because *Staphylococcus epidermidis* is a common skin commensal and potential contaminant, its isolation from pus specimens and the observed cefoxitin resistance rate among the 22 isolates should be interpreted with caution, particularly in the absence of detailed clinical correlation.

**Table 6:** Antimicrobial resistance profile of *Staphylococcus epidermidis* to commonly used antibiotics.

Antimicrobial agents	Resistance rate (%)	Susceptibility rate (%)	Intermediate rate (%)
Cefoxitin screening	68.2	31.8	0.0
Penicillin	90.9	9.1	0.0
Erythromycin	63.6	36.4	0.0
Clindamycin	13.6	81.8	4.5
Trimethoprim-sulfamethoxazole	9.1	90.9	0.0
Ciprofloxacin	9.1	90.9	0.0
Gentamicin	4.5	86.4	9.1
Vancomycin	0.0	100.0	0.0
Linezolid	0.0	100.0	0.0
Levofloxacin	31.8	68.2	0.0

#### 3.3.3 Streptococcus agalactiae

The antimicrobial susceptibility profile of *Streptococcus agalactiae* is shown in **Table 7**. All *Streptococcus agalactiae* isolates showed high susceptibility to the antimicrobial agents tested in this study. Specifically, susceptibility rates to penicillin, ampicillin, vancomycin, linezolid, levofloxacin, and benzathine penicillin were all 100.0%, with no resistant or intermediate isolates detected. Given the small number of *Streptococcus agalactiae* isolates (n = 8), these antimicrobial susceptibility results should be interpreted with caution and considered descriptive only.

**Table 7:** Antimicrobial resistance profile of *Streptococcus agalactiae* to commonly used antibiotics.

Antimicrobial agents	Resistance rate (%)	Susceptibility rate (%)	Intermediate rate (%)
Penicillin	0.0	100.0	0.0
Ampicillin	0.0	100.0	0.0
Vancomycin	0.0	100.0	0.0
Linezolid	0.0	100.0	0.0
Levofloxacin	0.0	100.0	0.0
Benzathine penicillin	0.0	100.0	0.0

## 4 Discussion

This study analyzed the distribution of pathogens and antimicrobial susceptibility profiles of pus culture specimens collected from hospitalized patients at Longgang District Maternity & Child Healthcare Hospital of Shenzhen City between 2021 and 2025. Gram-negative bacteria predominated among positive pus culture specimens, with *Escherichia coli* and *Staphylococcus aureus* identified as the two leading isolates. Descriptively, Gram-negative bacteria accounted for approximately 70% of isolates during 2023-2025, indicating that Gram-negative bacilli represented an important component of positive pus culture isolates in recent years.

This finding is not entirely consistent with some traditional assumptions. Previous understanding of skin and soft tissue infections, wound infections, and localized purulent lesions has often emphasized the importance of Gram-positive cocci, particularly *Staphylococcus aureus* (Pinchera et al., 2022). However, recent studies have repeatedly shown that Gram-negative bacilli, including *Escherichia coli*, *Klebsiella pneumoniae*, and *Pseudomonas aeruginosa*, also play important roles in postoperative infections, complex wounds, and healthcare-associated infections (Fakhreddine et al., 2026; Kachipedzu et al., 2024; Odoko et al., 2024). In other words, localized suppurative infections do not have a fixed pathogen spectrum; rather, their microbial composition is closely related to patient source, inpatient department structure, infection site, and the local hospital microbiological environment (Bilal et al., 2025; Qiao et al., 2025). The predominance of Gram-negative bacteria observed in this study suggests that, in the specific setting of a maternal and child healthcare specialty hospital, empirical antimicrobial therapy for localized suppurative infections should not focus primarily on Gram-positive cocci alone.

Recent studies from pediatric and maternal and child healthcare settings in China have shown that Gram-negative bacteria, particularly Enterobacterales such as *Escherichia coli* and *Klebsiella pneumoniae*, are important clinical isolates, and ESBL production remains a notable resistance concern (Li et al., 2024; Pan et al., 2024). Recent wound infection studies from China also reported mixed pathogen profiles involving *Staphylococcus aureus* and Gram-negative bacilli, with multidrug-resistant organisms remaining clinically relevant (Khan et al., 2025; Wang et al., 2024). Consistent with these findings, the predominance of Gram-negative bacteria and ESBL-producing isolates in our study suggests that Gram-negative pathogens warrant attention in pus-related infections in maternal and child healthcare specialty hospitals. The relatively higher proportion of Gram-negative bacteria observed during 2023–2025 may be related to changes in specimen distribution, departmental composition, perioperative practices, antimicrobial exposure, or local antimicrobial selection pressure; however, because detailed data were unavailable, this explanation remains speculative and requires further validation.

From the perspective of specimen source and patient composition, pus specimens in this study were derived mainly from Pediatric Surgery, General Surgery, Gynecology, and Breast Surgery, indicating that localized suppurative infections in our hospital arose from multiple clinical contexts rather than a single disease setting. Pediatric Surgery cases were concentrated in young children and showed a male predominance, particularly in the 0-3-year group, a pattern also reported in pediatric abscess cohorts, especially in pediatric perianal abscess (Ajaj et al., 2024; Ding et al., 2024). However, because cases were not further stratified by specific disease entity or infection site, this sex difference is better interpreted as a demographic feature than as evidence of a specific mechanism. Overall, the coexistence of young children and women of reproductive age contributed to a mixed pathogen spectrum, with staphylococcal infections remaining prominent because of the high proportion of Pediatric Surgery cases, while Enterobacterales were also important in gynecologic, general surgical, and perioperative infections. Accordingly, these findings are more representative of the overall microbiological features of localized suppurative infections in maternal and child healthcare specialty hospitals than of any single disease entity or infection site.

Among the major Gram-negative bacilli, *Escherichia coli* was not only the most common pathogen in this study but also the organism with the greatest resistance burden. The rate of extended-spectrum beta-lactamase production was 28.1%, and resistance rates to ampicillin, trimethoprim-sulfamethoxazole, ampicillin/sulbactam, and ciprofloxacin were 77.9%, 47.7%, 36.4%, and 28.6%, respectively, whereas susceptibility to imipenem and amikacin remained high. *Klebsiella pneumoniae subsp. pneumoniae* also showed a certain proportion of extended-spectrum beta-lactamase-positive isolates and had already developed some resistance to cefuroxime sodium, ceftriaxone, and ciprofloxacin, while retaining 100.0% susceptibility to amikacin, cefoxitin, and ertapenem. These findings are broadly consistent with recent reports on localized infections, which have shown that Enterobacterales commonly exhibit resistance to ampicillin, trimethoprim-sulfamethoxazole, and some fluoroquinolones, whereas amikacin and carbapenems usually retain good activity (Asaduzzaman et al., 2026; Wu et al., 2024).

From a clinical perspective, these findings have direct implications. For pus-related infections in our hospital, particularly in cases involving complex infection, prior antimicrobial exposure, prolonged hospitalization, or poor response to initial empirical therapy, ampicillin, trimethoprim-sulfamethoxazole, and some fluoroquinolones may no longer be appropriate as empirical first-line options. By contrast, carbapenems and amikacin may still have value in severe infections or infections caused by resistant organisms. This does not imply that the routine use of broad-spectrum agents should be expanded; rather, it underscores the need for empirical therapy to be more closely guided by local pathogen distribution and antimicrobial susceptibility data, thereby reducing overreliance on conventional assumptions (Pipitone et al., 2025).

Although *Pseudomonas aeruginosa* accounted for only 2.4% of isolates in this study, and its overall proportion was low, its clinical significance should not be underestimated. Our data showed that this organism remained highly susceptible to imipenem, meropenem, piperacillin/tazobactam, ceftazidime, cefepime, and amikacin, and showed some resistance to ciprofloxacin and levofloxacin. These findings suggest that *Pseudomonas aeruginosa* associated with pus infections in our hospital has not yet developed a widespread multidrug-resistant pattern. Nevertheless, because this organism is often encountered in complex wounds, prolonged hospitalization, recurrent treatment, and infections associated with invasive procedures, continued surveillance remains necessary, particularly among high-risk inpatients (Itani et al., 2025; Schwartz et al., 2024).

Among the Gram-positive cocci, *Staphylococcus aureus* remained the most important Gram-positive pathogen in this study. Resistance rates to cefoxitin screening and oxacillin were 25.0% and 22.0%, respectively, indicating that a certain proportion of pus isolates in our hospital were methicillin resistant. At the same time, resistance rates to erythromycin and clindamycin were as high as 48.5% and 47.7%, whereas susceptibility to vancomycin and linezolid remained high. These findings are consistent with the general trend reported in recent studies of localized infections, namely that *Staphylococcus aureus* remains one of the core pathogens in localized suppurative infections (Bazanelli Prebianchi et al., 2025; Li et al., 2025; Liu et al., 2024), that the burden of methicillin resistance and macrolide/lincosamide resistance deserves continued attention, and that vancomycin and linezolid usually retain good activity.

These findings suggest that, when a localized suppurative infection caused by staphylococci is suspected in our hospital, erythromycin and clindamycin should be used more cautiously in empirical therapy and should not be considered routine first-choice agents. By comparison, vancomycin and linezolid remain relatively reliable treatment options when resistant staphylococcal infection is more likely or when the clinical response is suboptimal. It should also be noted that, although the proportion of methicillin resistance in this study was not extremely high, it was sufficient to indicate that methicillin-resistant *Staphylococcus aureus* should not be overlooked in empirical treatment decisions.

Although *Staphylococcus epidermidis* accounted for only 3.8% of isolates in this study, its resistance profile remains noteworthy. The cefoxitin screening resistance rate reached 68.2%, which was higher than that of *Staphylococcus aureus*. High resistance rates were also observed for penicillin and erythromycin, whereas susceptibility to vancomycin and linezolid remained 100.0%. These findings are consistent with the general pattern that coagulase-negative staphylococci are more likely to exhibit methicillin-resistant phenotypes in hospital- and device-associated infection settings. Nevertheless, the pathogenic significance of *Staphylococcus epidermidis* in pus specimens still needs to be interpreted in conjunction with sampling method, infection site, and clinical presentation, and not every isolate should be regarded as a definite pathogen. Even so, in postoperative incisions, pericatheter infections, or recurrent infectious lesions, its resistance profile may still have practical reference value.

*Streptococcus agalactiae* was isolated infrequently in this study, accounting for only 1.4% of isolates, but given the maternal and child healthcare specialty setting of our hospital, its detection still carries some specialty-specific relevance. In this study, *Streptococcus agalactiae* showed 100.0% susceptibility to penicillin, ampicillin, vancomycin, linezolid, levofloxacin, and benzathine penicillin. This is broadly consistent with current understanding of the resistance characteristics of *Streptococcus agalactiae*, namely that overall susceptibility to penicillin remains high (Boyanov et al., 2024; Hsu et al., 2025; Mai et al., 2025). However, because the number of *Streptococcus agalactiae* isolates in this study was small, these findings are better regarded as supplementary information on the local pathogen spectrum rather than as evidence for firm conclusions regarding resistance trends.

Several limitations should be acknowledged. This was a single-center retrospective descriptive study based on routine laboratory information system data from hospitalized patients in a maternal and child healthcare specialty hospital. Therefore, the pathogen distribution and antimicrobial susceptibility profiles may have been influenced by the hospital’s patient population, departmental structure, specimen submission practices, and local antimicrobial formulary, and the findings should be interpreted mainly as local microbiological surveillance data rather than broadly generalized to other settings. The analysis was limited to routine aerobic pus cultures and did not include anaerobic or fungal cultures or molecular testing; therefore, obligate anaerobic bacteria commonly involved in abscesses, as well as other clinically relevant pathogens, may have been underestimated. Extended-spectrum *β*-lactamase production among Enterobacterales was determined using routine VITEK 2 Compact ESBL detection results, and additional manual phenotypic confirmatory testing was not routinely performed for all isolates. In addition, molecular detection of ESBL-related genes such as CTX-M, TEM, and SHV was not conducted. Therefore, the prevalence of ESBL-producing *Escherichia coli* and *Klebsiella pneumoniae* should be interpreted with these methodological limitations in mind. In addition, antimicrobial susceptibility interpretations were based on routine categorical results reported during the study period rather than retrospective re-interpretation of raw MIC values using a single CLSI edition; therefore, potential changes in CLSI breakpoints over time may have affected the comparability of susceptibility results across years. The D-test was not routinely performed for erythromycin-resistant, clindamycin-susceptible *Staphylococcus aureus* isolates; therefore, inducible macrolide-lincosamide-streptogramin B resistance could not be assessed, and clindamycin susceptibility should be interpreted with caution. Detailed clinical information, including infection site, prior antimicrobial exposure, comorbidities, treatment regimens, and outcomes, was not systematically available; thus, clinical risk factors, treatment responses, and prognostic associations could not be assessed. The small number of isolates from some departments and species may also limit subgroup interpretation. Further multicenter prospective studies integrating microbiological and clinical data are needed.

The practical significance of this study lies in showing that empirical treatment strategies for localized suppurative infections in maternal and child healthcare specialty hospitals cannot simply be adopted from general hospitals or from single-disease infection scenarios. In our hospitalized population, the pathogen spectrum of pus cultures showed a distinctly mixed pattern. On the one hand, the high proportion of Pediatric Surgery cases suggests that staphylococcal infections should remain an important target of empirical coverage. On the other hand, the presence of gynecologic, surgical, and perioperative cases highlights the increasing importance of Enterobacterales in localized infections. Therefore, empirical treatment should be stratified according to the specific clinical context. For typical purulent skin and soft tissue lesions, treatment regimens should include coverage for staphylococci, whereas for lower abdominal, perineal, postoperative incision, and complex healthcare-associated infections, the possible involvement of Enterobacterales should be carefully considered, with timely adjustment of antimicrobial therapy based on local pathogen distribution and antimicrobial susceptibility data.

## 5 Conclusion

Pus culture isolates from hospitalized patients at this maternal and child healthcare specialty hospital exhibited a distinct pathogen profile, with Gram-negative bacteria predominating. These findings suggest that empirical therapy for localized suppurative infections in this setting should be guided by local microbiological surveillance and tailored to the safety needs of children and women of reproductive age. Continued institution-specific monitoring may help optimize empirical treatment and support antimicrobial stewardship in this specialty population.

## Data Availability

The original contributions presented in the study are included in the article/supplementary material. Further inquiries can be directed to the corresponding authors.
